# Theory of Atomic-Scale Direct Thermometry Using Electron Spin Resonance via Scanning Tunneling Microscopy

**DOI:** 10.1021/acs.nanolett.4c05018

**Published:** 2025-02-02

**Authors:** Yelko del Castillo, Joaquín Fernández-Rossier

**Affiliations:** † International Iberian Nanotechnology Laboratory (INL), Av. Mestre José Veiga, 4715-330 Braga, Portugal; ‡ Centro de Física das Universidades do Minho e do Porto, Universidade do Minho, Campus de Gualtar, 4710-057 Braga, Portugal

**Keywords:** scanning tunneling microscopy, electron spin resonance, thermometry, thermal gradients, nanomagnetism, surface spins

## Abstract

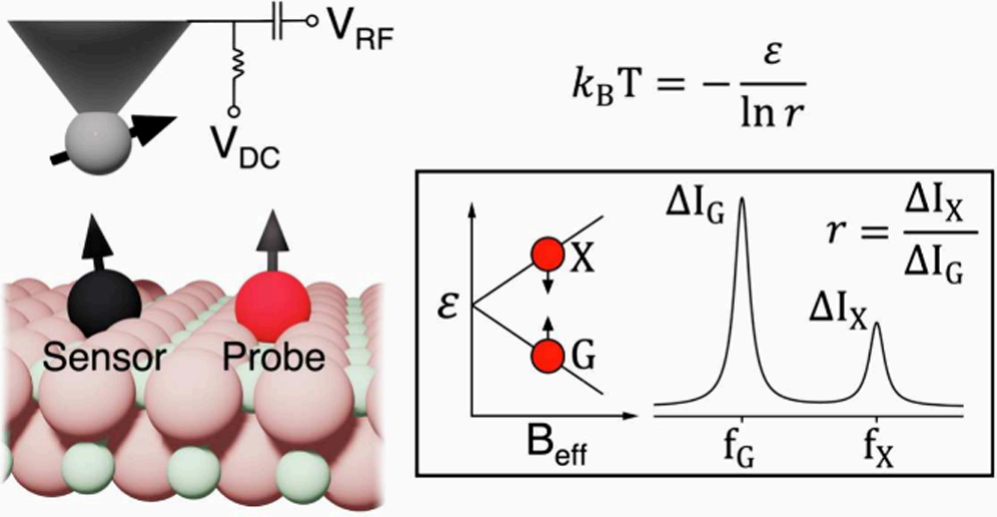

Knowledge of the occupation ratio and energy splitting of a two-level system provides a direct method for temperature readout. This principle was demonstrated for an individual two-level magnetic atom using Electron Spin Resonance via Scanning Tunneling Microscopy (ESR-STM). The temperature determination involves two steps: measuring the energy splitting with ESR-STM and determining the equilibrium occupation of a nearby atom using the peak height ratio in the ESR spectrum. Here we present a theory addressing three aspects: the impact of shot noise and back-action on thermometry precision, the role of spin geometry in enhancing signal-to-noise ratio, and the method’s capability to detect thermal gradients as small as 5 mK/nm. We predict ESR-STM thermometry achieves 10 mK resolution at around 1 K temperatures, offering new avenues for nanoscale thermal measurements.

Temperature measurements play a central role in many fields of science and technology.^1^ A large variety of techniques are used to measure temperature, that depend on different physical phenomena and are better suited for different ranges. These include resistance thermometry, that exploits the predictable change of electrical resistance with temperature of materials, such as platinum,^1^ magnetic thermometers that use the Curie–Weiss law,^2^ noise thermometry that relies on the well-established scaling of Johnson-Nyquist noise with temperature,^3,4^ nuclear quadrupole resonance thermometry,^5^ etc. A subclass of these methods is specifically designed to measure temperature with nanoscale probes,^6^ aiming to map temperature with very high spatial resolution, with important applications for efficient heat management in nanoelectronics^7^ and in biomedical applications.^8^ Important nanoscale thermometers include NV center magnetometry, which exploits the shift of resonance frequency due to thermal expansion,^9^ single-molecule chromophores, exploiting the temperature dependence of the photoluminescence line width,^10^ superconducting scanning tunnel microscope (STM) junctions^11^ and scanning thermal magnetometry.^7^ Yu-Shiba-Rusinov states have also been proposed as a potential method for nanoscale thermometry, using the sensitivity of the switching current to quantum phase transitions.^12^

All of these methods infer temperature by exploiting the well-known relation between a given physical property (resistance, resonance frequency, magnetization, noise) and temperature. Therefore, it can be said that they are *indirect*. In contrast, a direct determination of temperature would rely on its definition in terms of the average energy of particles in a system. In the context of a quantum system with two energy levels, *G* and *X*, temperature is defined in terms of the ratio of their occupations, following the Gibbs-Boltzmann equation:
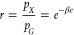
1where ε ≡ ε_*X*_ – ε_*G*_. Therefore, measurement of both ε and *r* would directly yield:
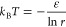
2thereby providing a *direct* measurement of temperature^1^, based on its definition. Building on the experimental work of Choi et al.,^13^ here we provide a theory for the resolution and the range of the method, and we also go beyond the original work by proposing an extension to measure thermal gradients.

The work of Choi et al.^13^ relies on ESR-STM (Electron Spin Resonance with Scanning Tunneling Microscopy)^14−24^ in a lateral sensing scheme to determine the temperature of individual magnetic atoms or molecules placed on a surface, whose location is determined with sub-Ångström resolution. Therefore, this method vastly outperforms the spatial resolution of any other scanning thermal microscopy techniques.^7^

The lateral sensing scheme entails an ESR-STM active surface spin, either an atom or a molecule, that we shall call the sensor spin (black atom under the STM tip in Figure 1a), placed nearby a second spin or group of spins, that we shall call the probe spin(s) (red atom in Figure 1a). The resonance spectrum of the sensor spin features several resonance peaks (see Figure 1b), instead of only one, on account of the dipolar coupling to the probe spins. The relative intensity of the different sensor resonance peaks reflects the relative probability for a given probe spin state to be occupied. Experiments^13^ have shown that the occupation of the probe states follows a thermal distribution, in contrast with that of the sensor atom, governed by the ESR-STM drive. This observation provides strong evidence of a very reduced back action of the sensor spin on the occupation of the probe-spin states, essential in the following discussion.

**Figure 1:**
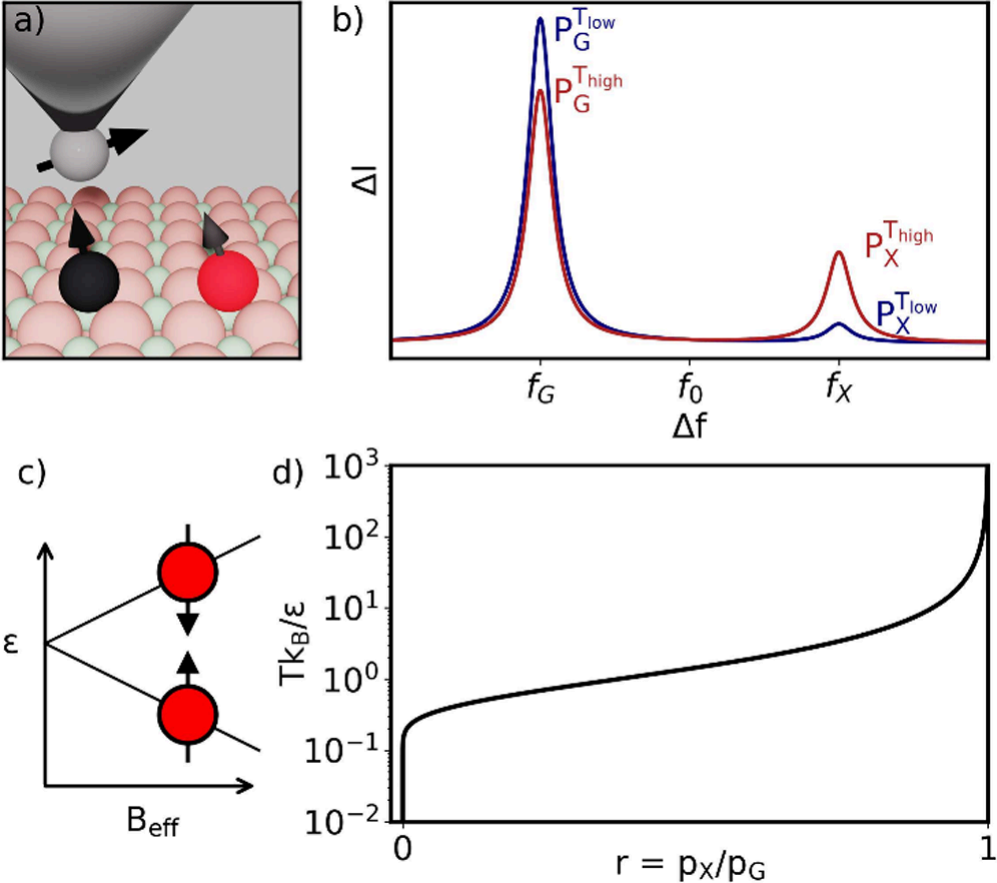
a) Scheme of the proposed atom arrangement to obtain the temperature. An STM tip over the sensor atom under the influence of the stray field generated by a nearby atom. b) ESR spectrum showing two resonance peaks corresponding to each state of the nearby atom for two different temperatures and how the ratio between the heights of the peaks changes. c) Energy splitting, ε, between the ground and excited states of the probe atom. d) Temperature dependence of the ratio between the up and down state peaks.

We first discuss the simplest case where an ESR-STM active surface spin is coupled to an individual *S* = 1/2 surface spin (see Figure 1a). We assume that the external magnetic field is dramatically larger than the dipolar and exchange interactions between the ESR-STM active sensor spin and the probe spin, whose temperature is being determined, so that the spin-flip terms in the Hamiltonian are negligible:
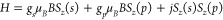
3where *s* and *p* stand for sensor and probe spin, respectively. In the Supporting Information, we show that this approximation is very good as long as the Zeeman splitting is much larger than the dipolar interaction. Specifically, we show that the fidelity of the Ising states with the exact eigenstates is larger than 99% for *B* > 0.4 T for two spins separated by 1 nm with *g* = 2 and *g* = 1.9. Then, we can write the sensor Hamiltonian as
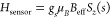
4where
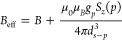
5Since the effective field can take 2 values, depending on whether the probe spin is in the state up or down, the ESR-STM spectrum for the sensor spin has two resonance peaks (see Figure 1c and b respectively). Experiments^13^ show that the ESR-STM spectra of the sensor spin relate to the occupation of the states of the probe spins, according to the equation
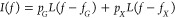
6where  are the thermal occupations of each state and *L*(*f* – *f*_*G*,*X*_) is a Lorentzian type resonance curve centered around the frequency *f*_*G*,*X*_. Assuming that the external field and the stray field of the probe atom only have perpendicular components, *f*_*G*,*X*_ is given by
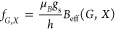
7where *h* = 2*πℏ*, and *g*_s_ is the gyromagnetic factor of the sensor.

Equation 2 relates the temperature to two quantities, the energy splitting of the probed spin, ε, and the ratio of the heights of the peaks in the ESR-STM spectrum of the sensor spin, *r*. These quantities have to be determined independently. The ratio *r* is obtained from the ESR-STM spectrum of the sensor atom. The most optimal determination of ε would be carried out using ESR-STM on the probe spin, as we discuss below, given the very good spectral resolution of this method, compared to inelastic electron tunnel spectroscopy (IETS). The precision of the determination of temperature using this method is thus limited by the precision to determine ε and *r*.
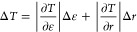
8

After derivation and rearrangement of the resulting equation and using the relation^25^, we arrive at the following expression (see Supporting Information):
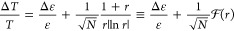
9where  is the number of tunneling electrons during the measurement time Δ*t*. A more transparent expression is obtained if we use *βϵ* instead of *r*:
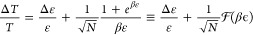
10We now assume that  is determined with ESR-STM. There are two sources of error for the determination of ε, that we label as intrinsic and back-action. The intrinsic sources of error relate to the experimental measurement of ε obtained in the absence of back-action by performing a spin resonance measurement on the isolated probe atom (if this one is ESR active). Using the result from,^26^ the shot-noise limited expression for the absolute error in ε is
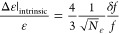
11where δ*f* is the width of the ESR-STM spectrum resonance peak and *N*_ε_ is the number of tunneling electrons during the measurement of ε, that may or may not be the same as *N* in eq 9. The intrinsic contribution to the error of ε has a prefactor controlled by the quality factor of the resonance,  for which values smaller than 10^–4^ have been reported.^14^ Therefore, we anticipate that the contribution of eq 11 is going to be negligible.

We now discuss the role of the back-action of the sensor spin on the occupation of the probe spins. Even if flip-flop interactions are blocked, on account of the small ratio between their magnitude and the energy difference of the states *G*_*s*_*X*_*p*_ and *X*_*s*_*G*_*p*_, the Ising interaction *jS*_*z*_(*s*)*S*_*z*_(*p*) is still active. Therefore, the effective field of the probe spin is given by the sum of the external field and the stray field created by the sensor spin, that depends on the average magnetization of the sensor spin,⟨*S*_*z*_(*s*)⟩, i.e., *B*_eff,p_ = *B* + *B*_stray_(⟨*S*_*z*_(*s*)⟩) At resonance, and in the limit *T*_1_*T*_2_Ω^2^ ≫ 1, we have ⟨*S*_*z*_(*s*)⟩ → 0,^27^ where *T*_1_, *T*_2_, Ω are the spin relaxation time, spin decoherence time and Rabi driving force of the sensor spin (see Supporting Information). However, given that the resonance value δ = 0 can only be achieved in a fraction of the instances, on account of the fact that δ depends on the state in which the probe spin is, the effective field of the probe spin is *definitely different* from the external field by an amount *smaller* than . We can therefore provide the following bound to ε
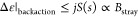
12taking the most pessimistic assumption that the average spin of the sensor is maximal. The relative error is given by the ratio of the external field and the stray field:
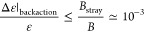
13Therefore, as anticipated, the back-action error is clearly larger than the intrinsic shot-noise limit (eq 11) when it comes to determining Δε. The Supporting Information shows the tip field effect error^28^ is comparable to the back-action error.

We now assess the magnitude of the shot-noise limit of Δ*r* (second term in eq 9) and compare it to the back-action error (eq 13). Figure 2a shows the function  controlling Δ*r* has a minimum  at *r* ≈ 0.28 (*βε* ≈ 1.28) and grows rapidly, diverging for *r* → 0 and *r* → 1 (see inset). Thus, the operational range of ESR-STM thermometry is around *r* ≃ 0.28, where  stays small.

**Figure 2:**
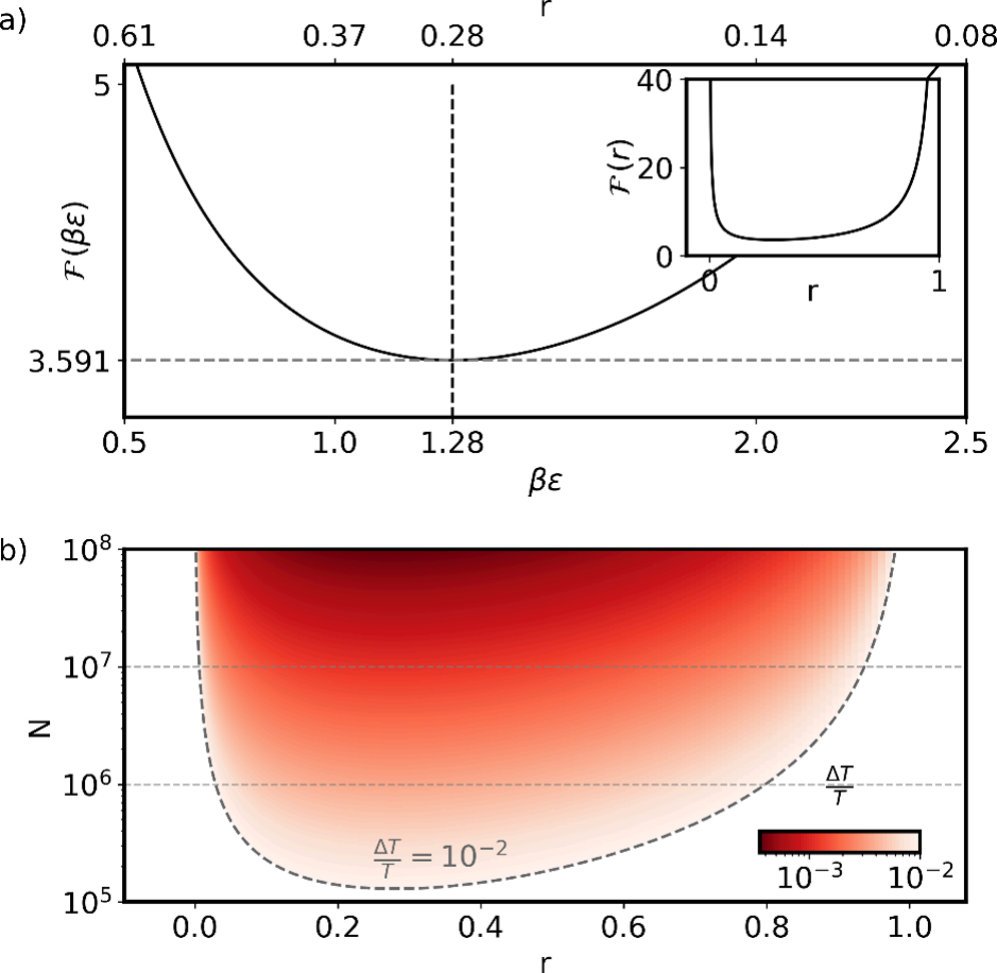
a) Numerical analysis of the minimal value of the second term in eq 9 as a function of *r* (top axis in red) and analogously for eq 10 as a function of *βε*. Inset shows the same result for the complete range of *r*, from 0 to 1. b) Range of *r* where the relative error in temperature is at least 10^–2^ or lower as a function of *N*.

We now estimate the minimal and maximal temperatures that could be measured, if we impose an upper bound to the second contribution to error in eq 8, . For this estimate, we assume Δ*t* ≈ 2 *s* and Δ*I* = 100 fA,^14,29−31^ leading to *N* ≈ 1.25 × 10^6^. We find the minimal and maximal values of *βε* are 0.2 and 3.7, respectively. We note that the operational range of the ESR-STM thermometer depends on the applied magnetic field. Assuming an applied field of 1 T, standard in the ESR-STM,^13,14,16,30,31^ and *g* = 2, we have ε = 115.7 μeV, so that the measurable temperature range with a relative error of  is between 0.36 and 6.77 K. In contrast, if ESR-STM is carried out at 0.1 T,^32^ our upper bound for  is satisfied for temperatures between 36 and 677 mK. However, this increases the back-action error (eq 13).

In summary, three factors limit ESR-STM thermometry resolution. The most significant is the back-action error from the sensing spin’s stray field on the probe spin, causing stochastic changes in the energy splitting ε (eq 2). Shot-noise limits peak-height ratio determination. This error can be made smaller than the back-action error by increasing the measurement time, via the  prefactor. This error sets the ESR-STM thermometer’s operational range due to the temperature dependence of  (Figure 2b). Finally, the resolution limit to determine ε is negligible, compared to the other two.

We now discuss additional limitations of the ESR-STM thermometry. First, the Zeeman splitting ε of the isolated probe atom is bounded by the maximum driving frequency of ESR-STM. Current ESR-STM experiments reach ∼100 GHz.^33^ For a spin-1/2 sensor with one μ_*B*_, this corresponds to a maximum external magnetic field of 3.6 T and an energy splitting of 413.6 μeV. Two factors limit peak height ratio determination for the probe atom. First, the magnitude of the peak splitting, determined by the magnitude of the stray field at the sensor spin, has to be larger than the peak line width, so that two peaks can be resolved. Consequently, this sets an upper limit to the separation of the sensor and the probe atoms. Shot noise limits the smallest variation of peak heights that can be resolved.^26^ Whereas shot noise can be theoretically reduced by increasing the measurement time, thermal drift of the STM position also sets an upper limit for this quantity. Displacements on the order of picometers per hour have been reported.^13^ This would lead to changes in the stray field and therefore a shift of the resonance peak. Finally, another degree of freedom to consider is the contribution of the stray field generated by the tip, where this one can have a very large magnetic moment. Although the stray field decays with the cube of the distance and can be thoroughly studied, it could vary in magnitude and direction depending on different external magnetic fields.

We now discuss how having  probe spins symmetrically placed around a sensor spin can be used to reduce shot noise in temperature readout. This concept was implemented by Choi and co-workers.^34^ First, we discuss the idealized case where the  probe spins do not interact with each other and have the same temperature, and their stray fields at the sensor spin are identical.

The ratio between the two lower energy peaks, the ground state, and the one corresponding to flipping one spin (*n* = 0 and *n* = 1, respectively) is given by the following expression (see Supporting Information):
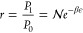
14From here, we derive the temperature equation:
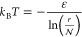
15

Analogously to the derivation of eq 9, for  identical probe spins we find
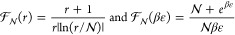
16For the simplest case of an engineered structure with , a sensor is placed equidistant between two identical atoms. We can label the four states of the probe atoms as *GG*, *GX*, *XG* and *XX*, where *G* and *X* stand for the ground and excited state, respectively. The complete ESR spectrum consists of three peaks: two lateral peaks, for states *GG* and *XX*, and a central peak at the resonance frequency of the isolated sensor atom that corresponds to the states *GX* and *XG*, whose stray fields cancel each other. The height of the central peak is proportional to the joint probability of the two possible states, resulting in almost twice the change in height for the same temperature variation compared to a single-atom readout.

This improvement in the variation of the peak with temperature translates to a reduced contribution to the Δ*r* noise (second term in eq 8). As shown in Figure 3b, compared to a single atom, for , the measurable temperature range within the divergence points of  is approximately 1.5 times larger, and the minimum relative error is about two-thirds.

**Figure 3:**
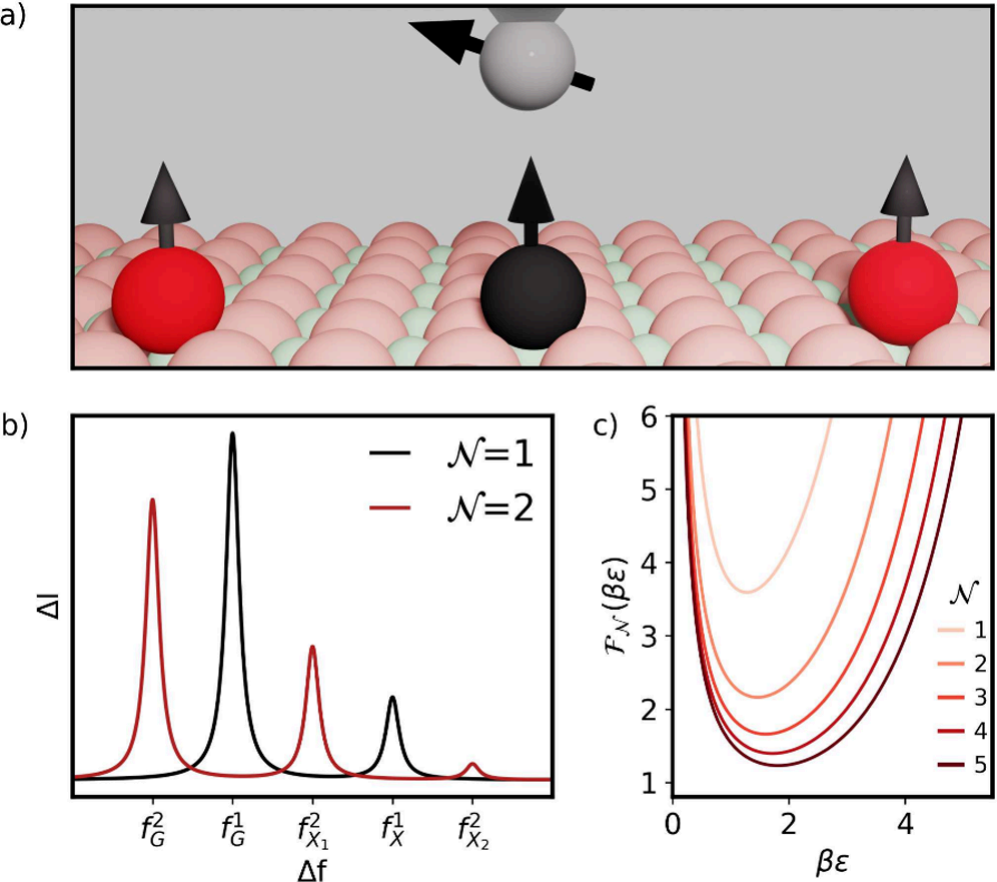
a) Proposed arrangement using  identical probe spins to improve the range and precision of measurements. b) Comparison between the ESR spectrum using one and two probe spins. c) Minima of  as a function of .

In Figure 3b and c, we show how the *βε* range allowed for measurement and the minimum presented in eq 9, respectively, improve with . We observe that the improvement saturates and, for most practical scenarios,  is sufficient.

We now propose a method to probe thermal gradients in two atoms, extending the ESR-thermometry to the case where temperatures are not homogeneous. We consider an ESR active sensor spin placed between two identical two-level magnetic atoms, that we label as *a* and *b*, whose temperature difference *T*_*a*_ – *T*_*b*_ we want to determine (see Figure 4a). The thermal gradient is given by
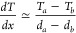
17where *d*_*a*_ and *d*_*b*_ are the positions of *a* and *b* atoms along the *x* axis in the plane of the surface. The atoms *a* and *b* are separated by a distance in the range of 2–4 nm. This distance is limited by the range of the stray field created by a point magnetic moment and the spectral sensitivity of the ESR active spin. The values of *d*_*a*_ and *d*_*b*_ can be determined using STM with subatomic resolution. Therefore, the nontrivial part of the thermal gradient measurement is the determination of the thermal difference *T*_dif_ ≡ *T*_*a*_ – *T*_*b*_. To do so, we propose to place the sensor atom in the line that joins atoms *a* and *b*, but closer to one of the probe atoms. This differs from the geometry considered in Figure 3a, where the sensor atom was equidistant from the outer atoms. As a result, instead of a single central peak that corresponds to the *XG* and *GX* configurations, there are two peaks, as the stray fields no longer compensate each other (see Figure 4b) and a total of four peaks. Since we assume again that the magnetic moments of the spins are along the *z*-axis, perpendicular to the surface, the external and stray fields add up, and the frequencies of the four resonant peaks are given by
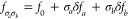
18

**Figure 4:**
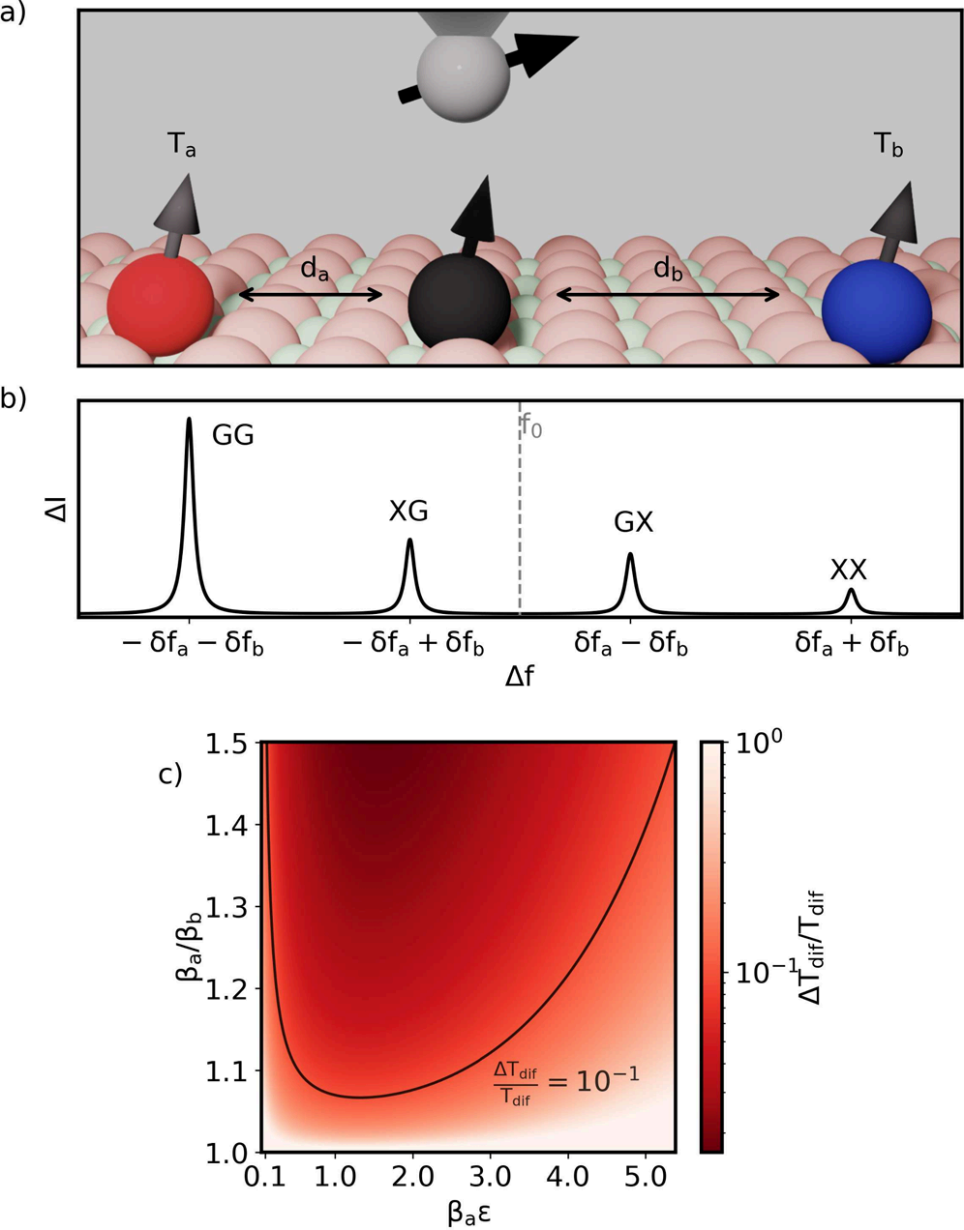
a) Scheme proposed to measure thermal gradient. A sensor atom is placed nonequidistantly between two atoms with different temperatures. b) ESR spectrum resulting from the atom arrangement in panel a. c) Relative error of the thermal difference as a function of both the temperature in one spin and the difference between both probe spins for *N* = 1.25 × 10^6^.

The determination of the thermal difference is very similar to the determination of temperature. We first realize that we can easily relate the temperature variation to the exponential factors that appear in Boltzmann distributions:
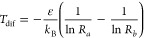
19where  and . We next relate *R*_*a*_ and *R*_*b*_ to the ratios of the heights of the peaks in the ESR-STM spectrum of the sensor atom. To derive the heights of the peaks of the ESR-STM spectra, we assume that Zeeman energy is much larger than dipolar (or exchange) coupling between atoms *a* and *b*. As a result, the energy spectra are additive, , . Since the atoms are identical, we take ε_*a*_ = ε_*b*_ = ε. We can now write the Boltzmann factors for the states *GG*, *GX*, *XG* as products of probabilities of atoms *a* and *b*:
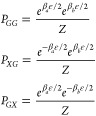
20where *Z* is the partition function. From here we obtain right away:
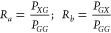
21

Therefore, the determination of the temperature difference is then carried out from the readout of the ratios of the two central peaks in the ESR spectra with the ground state (*GG*) peak. The analysis of the resolution of the proposed ESR-STM thermal gradient measurement runs parallel to the one of the ESR-STM thermometer, discussed above. Analogously to eqs 9 and 10 we find the following expression for the temperature difference resolution (See Supporting Information):
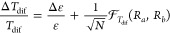
22where
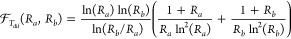
23and *N* is the number of tunneling electrons recorded during the measurement time. The first contribution to the error is dominated by back-action error (see eq 13). The second contribution, associated with the shot-noise limit in the determination of peak/height ratios, is shown in Figure 4c, as a function of the temperature of spin *a* (β_*a*_ε) and the temperature ratio of atoms *b* and *a* (β_*a*_/β_*b*_) We assume *N* = 1.25 × 10^6^, that corresponds to a measurement time of approximately 2 s, with a current of 100 fA. The solid line in the figure corresponds to a shot-noise relative error of 10%. From our numerics, the shot-noise error is never smaller than 1% (for the assumed value of *N*), and therefore, larger than the first term in eq 22. We identify an optimal temperature range, centered around β_*a*_ε ≈ 1.28, where the relative error is minimized. The relative error also increases as the temperature difference between the atoms decreases.

To give an example of the operational temperature range of the STM-ESR temperature gradient measurement, we assume that atoms *a* and *b* have *g* = 2, *S* = 1/2 and *B* = 1*T*, so that ε = 115.7 μeV. We set Δ*T*_dif_/*T*_dif_ = 10^–1^ as the upper bound for the relative shot-noise error. With a 10% temperature difference (β_*a*_/β_*b*_ = 1.1), the operational temperature range is between 0.5 and 2.5 K. Therefore, for *T*_*a*_ = 1 K and *T*_*b*_ = 1.1 K, this method could achieve a resolution of 10 mK. Thermal gradients of 0.74 K across a distance of 15 nm, equivalent to ≃50 mK in nm, have been reported experimentally.^35^

Considering an ESR-STM resonance width of 3.6 MHz,^14^ atoms with a magnetic moment of 1 μ_*B*_ can be positioned 2 nm apart, generating sufficient signal on the sensor to distinguish the central peaks (see Supporting Information) and determine the ratio. This dramatically outperforms scanning thermal magnetometry,^7^ for which lateral resolutions are in the range of 100 nm. Combined with the 10 mK temperature difference relative error, ESR-STM offers a potential gradient resolution of  mK/nm.

In conclusion, we have analyzed the working principles and the resolution limits of the ESR-STM quantum sensing technique to carry out direct temperature and temperature gradient measurements. We have emphasized that, unlike most temperature determination methods, ESR-STM provides a direct measurement of temperature, relying directly on the Boltzmann formula that relates temperature, energy and average occupation of quantum state. We have quantified the limits that both shot-noise and back-action set to the resolution and operational range of the methods, and we find that they dramatically outperform alternative techniques. Our theory relies on the lateral-sensing ESR-STM scheme that requires the resonant spin to be placed on an MgO surface, the standard situation so far.^14−24^ However, the implementation of ESR-STM with the resonating spin placed in the STM tip, recently reported,^36^ holds the promise of extending the application of the technique to any conducting surface, and therefore, opens the door for the implementation of direct temperature measurements with atomic-scale resolution, and may advance our understanding of many physical phenomena, including radiative heat transfer at the nanoscale.^37^

## Supporting Information Available

An estimation of the simplified Hamiltonian validity; a derivation of the relative error for the temperature measurement; a discussion of the back-action; an estimation of the tip field effect; a derivation of the ratio expression using engineered structures; and a derivation of the relative error of the temperature difference (PDF)

## Footnotes

1 Or more precisely, temperature times the Boltzmann constant *k*_*B*_

## References

[ref1] Childs P. R.; Greenwood J.; Long C. Review of temperature measurement. Review of scientific instruments 2000, 71, 2959–2978. 10.1063/1.1305516.

[ref2] Dellby B.; Ekstrom H. A magnetic susceptibility balance for use in the temperature range 1.6–300 K. Journal of Physics E: Scientific Instruments 1971, 4, 342 10.1088/0022-3735/4/5/002.

[ref3] White D.; Galleano R.; Actis A.; Brixy H.; De Groot M.; Dubbeldam J.; Reesink A.; Edler F.; Sakurai H.; Shepard R.; et al. The status of Johnson noise thermometry. Metrologia 1996, 33, 325 10.1088/0026-1394/33/4/6.

[ref4] Qu J.; Benz S.; Rogalla H.; Tew W.; White D.; Zhou K. Johnson noise thermometry. Measurement Science and Technology 2019, 30, 112001 10.1088/1361-6501/ab3526. PMC11194799 38915953

[ref5] Utton D. Nuclear quadrupole resonance thermometry. Metrologia 1967, 3, 98 10.1088/0026-1394/3/4/002.

[ref6] Brites C. D.; Lima P. P.; Silva N. J.; Millán A.; Amaral V. S.; Palacio F.; Carlos L. D. Thermometry at the nanoscale. Nanoscale 2012, 4, 4799–4829. 10.1039/c2nr30663h. 22763389

[ref7] Zhang Y.; Zhu W.; Hui F.; Lanza M.; Borca-Tasciuc T.; Muñoz Rojo M. A review on principles and applications of scanning thermal microscopy (SThM). Adv. Funct. Mater. 2020, 30, 1900892 10.1002/adfm.201900892.

[ref8] Aslam N.; Zhou H.; Urbach E. K.; Turner M. J.; Walsworth R. L.; Lukin M. D.; Park H. Quantum sensors for biomedical applications. Nature Reviews Physics 2023, 5, 157–169. 10.1038/s42254-023-00558-3. 36776813 PMC9896461

[ref9] Fukami M.; Yale C.; Andrich P.; Liu X.; Heremans F.; Nealey P.; Awschalom D. All-optical cryogenic thermometry based on nitrogen-vacancy centers in nanodiamonds. Physical Review Applied 2019, 12, 014042 10.1103/PhysRevApplied.12.014042.

[ref10] Esteso V.; Duquennoy R.; Ng R. C.; Colautti M.; Lombardi P.; Arregui G.; Chávez-Angel E.; Sotomayor-Torres C.; Garcia P.; Hilke M.; et al. Quantum Thermometry with Single Molecules in Nanoprobes. PRX Quantum 2023, 4, 040314 10.1103/PRXQuantum.4.040314.

[ref11] Esat T.; Yang X.; Mustafayev F.; Soltner H.; Tautz F. S.; Temirov R. Determining the temperature of a millikelvin scanning tunnelling microscope junction. Communications Physics 2023, 6, 81 10.1038/s42005-023-01201-4.

[ref12] Karan S.; Huang H.; Padurariu C.; Kubala B.; Theiler A.; Black-Schaffer A. M.; Morrás G.; Yeyati A. L.; Cuevas J. C.; Ankerhold J.; et al. Superconducting quantum interference at the atomic scale. Nat. Phys. 2022, 18, 893–898. 10.1038/s41567-022-01644-6.

[ref13] Choi T.; Paul W.; Rolf-Pissarczyk S.; Macdonald A. J.; Natterer F. D.; Yang K.; Willke P.; Lutz C. P.; Heinrich A. J. Atomic-scale sensing of the magnetic dipolar field from single atoms. Nat. Nanotechnol. 2017, 12, 420–424. 10.1038/nnano.2017.18. 28263962

[ref14] Baumann S.; Paul W.; Choi T.; Lutz C. P.; Ardavan A.; Heinrich A. J. Electron paramagnetic resonance of individual atoms on a surface. Science 2015, 350, 417–420. 10.1126/science.aac8703. 26494753

[ref15] Yang K.; Bae Y.; Paul W.; Natterer F. D.; Willke P.; Lado J. L.; Ferrón A.; Choi T.; Fernández-Rossier J.; Heinrich A. J.; et al. Engineering the eigenstates of coupled spin-1/2 atoms on a surface. Phys. Rev. Lett. 2017, 119, 227206 10.1103/PhysRevLett.119.227206. 29286811

[ref16] Willke P.; Bae Y.; Yang K.; Lado J. L.; Ferrón A.; Choi T.; Ardavan A.; Fernández-Rossier J.; Heinrich A. J.; Lutz C. P. Hyperfine interaction of individual atoms on a surface. Science 2018, 362, 336–339. 10.1126/science.aat7047. 30337408

[ref17] Natterer F. D.; Patthey F.; Bilgeri T.; Forrester P. R.; Weiss N.; Brune H. Upgrade of a low-temperature scanning tunneling microscope for electron-spin resonance. Rev. Sci. Instrum. 2019, 10.1063/1.5065384. 30709206

[ref18] Seifert T. S.; Kovarik S.; Juraschek D. M.; Spaldin N. A.; Gambardella P.; Stepanow S. Longitudinal and transverse electron paramagnetic resonance in a scanning tunneling microscope. Science Advances 2020, 6, eabc5511 10.1126/sciadv.abc5511. 32998882 PMC7527223

[ref19] van Weerdenburg W. M.; Steinbrecher M.; van Mullekom N. P.; Gerritsen J. W.; von Allwörden H.; Natterer F. D.; Khajetoorians A. A. A scanning tunneling microscope capable of electron spin resonance and pump–probe spectroscopy at mK temperature and in vector magnetic field. Rev. Sci. Instrum. 2021, 10.1063/5.0040011. 33820009

[ref20] Farinacci L.; Veldman L. M.; Willke P.; Otte S. Experimental determination of a single atom ground state orbital through hyperfine anisotropy. Nano Lett. 2022, 22, 8470–8474. 10.1021/acs.nanolett.2c02783. 36305860 PMC9650725

[ref21] Kovarik S.; Robles R.; Schlitz R.; Seifert T. S.; Lorente N.; Gambardella P.; Stepanow S. Electron paramagnetic resonance of alkali metal atoms and dimers on ultrathin MgO. Nano Lett. 2022, 22, 4176–4181. 10.1021/acs.nanolett.2c00980. 35512394

[ref22] Zhang X.; Wolf C.; Wang Y.; Aubin H.; Bilgeri T.; Willke P.; Heinrich A. J.; Choi T. Electron spin resonance of single iron phthalocyanine molecules and role of their non-localized spins in magnetic interactions. Nat. Chem. 2022, 14, 59–65. 10.1038/s41557-021-00827-7. 34764471

[ref23] Kot P.; Ismail M.; Drost R.; Siebrecht J.; Huang H.; Ast C. R. Electric control of spin transitions at the atomic scale. Nat. Commun. 2023, 14, 6612 10.1038/s41467-023-42287-2. 37857623 PMC10587172

[ref24] Wang Y.; Chen Y.; Bui H. T.; Wolf C.; Haze M.; Mier C.; Kim J.; Choi D.-J.; Lutz C. P.; Bae Y.; et al. An atomic-scale multi-qubit platform. Science 2023, 382, 87–92. 10.1126/science.ade5050. 37797000

[ref25] del Castillo Y.; Fernández-Rossier J. Certifying entanglement of spins on surfaces using ESR-STM. Phys. Rev. B 2023, 108, 115413 10.1103/PhysRevB.108.115413.

[ref26] del Castillo Y.; Fernández-Rossier J. Probing spin fractionalization with electron spin resonance based on scanning tunneling microscopy. Phys. Rev. B 2024, 110, 045145 10.1103/PhysRevB.110.045145.

[ref27] Delgado F.; Fernández-Rossier J. Spin decoherence of magnetic atoms on surfaces. Prog. Surf. Sci. 2017, 92, 40–82. 10.1016/j.progsurf.2016.12.001.

[ref28] Yang K.; Paul W.; Natterer F. D.; Lado J. L.; Bae Y.; Willke P.; Choi T.; Ferrón A.; Fernández-Rossier J.; Heinrich A. J.; et al. Tuning the exchange bias on a single atom from 1 mT to 10 T. Phys. Rev. Lett. 2019, 122, 227203 10.1103/PhysRevLett.122.227203. 31283288

[ref29] Willke P.; Paul W.; Natterer F. D.; Yang K.; Bae Y.; Choi T.; Fernández-Rossier J.; Heinrich A. J.; Lutz C. P. Probing quantum coherence in single-atom electron spin resonance. Science Advances 2018, 4, eaaq1543 10.1126/sciadv.aaq1543. 29464211 PMC5815865

[ref30] Natterer F. D.; Yang K.; Paul W.; Willke P.; Choi T.; Greber T.; Heinrich A. J.; Lutz C. P. Reading and writing single-atom magnets. Nature 2017, 543, 226–228. 10.1038/nature21371. 28277519

[ref31] Willke P.; Singha A.; Zhang X.; Esat T.; Lutz C. P.; Heinrich A. J.; Choi T. Tuning single-atom electron spin resonance in a vector magnetic field. Nano Lett. 2019, 19, 8201–8206. 10.1021/acs.nanolett.9b03559. 31661282

[ref32] Steinbrecher M.; Van Weerdenburg W. M.; Walraven E. F.; Van Mullekom N. P.; Gerritsen J. W.; Natterer F. D.; Badrtdinov D. I.; Rudenko A. N.; Mazurenko V. V.; Katsnelson M. I.; et al. Quantifying the interplay between fine structure and geometry of an individual molecule on a surface. Phys. Rev. B 2021, 103, 155405 10.1103/PhysRevB.103.155405.

[ref33] Drost R.; Uhl M.; Kot P.; Siebrecht J.; Schmid A.; Merkt J.; Wünsch S.; Siegel M.; Kieler O.; Kleiner R. Combining electron spin resonance spectroscopy with scanning tunneling microscopy at high magnetic fields. Rev. Sci. Instrum. 2022, 10.1063/5.0078137. 35489929

[ref34] Choi D.-J.; Lorente N.; Wiebe J.; von Bergmann K.; Otte A. F.; Heinrich A. J. Colloquium: Atomic spin chains on surfaces. Rev. Mod. Phys. 2019, 91, 041001 10.1103/RevModPhys.91.041001.

[ref35] Hoffmann E. A.; Nilsson H. A.; Matthews J. E.; Nakpathomkun N.; Persson A. I.; Samuelson L.; Linke H. Measuring temperature gradients over nanometer length scales. Nano Lett. 2009, 9, 779–783. 10.1021/nl8034042. 19159269

[ref36] Esat T.; Borodin D.; Oh J.; Heinrich A. J.; Tautz F. S.; Bae Y.; Temirov R. A quantum sensor for atomic-scale electric and magnetic fields. Nat. Nanotechnol. 2024, 19, 1466 10.1038/s41565-024-01724-z. 39054385 PMC11486657

[ref37] Cuevas J. C.; García-Vidal F. J. Radiative heat transfer. Acs Photonics 2018, 5, 3896–3915. 10.1021/acsphotonics.8b01031.

