# Supplementary material for: Theory of Atomic-Scale Direct Thermometry Using Electron Spin Resonance via Scanning Tunneling Microscopy
Source: Nano Lett. 2025 Feb 2;25(6):2159–65. doi: 10.1021/acs.nanolett.4c05018 (PMC11827104; doi:10.1021/acs.nanolett.4c05018)
Supplement: Supplementary file 1 — nl4c05018_si_001.pdf [file nl4c05018_si_001.pdf]

Theory of atomic-scale direct thermometry using ESR-STM: Supporting Information

Yelko del Castillo[†,‡] and Joaquín Fernández-Rossier [*,†,¶]

†*International Iberian Nanotechnology Laboratory (INL), Av. Mestre José Veiga, 4715-330 Braga, Portugal*

‡*Centro de Física das Universidades do Minho e do Porto, Universidade do Minho, Campus de Gualtar, 4710-057 Braga, Portugal*

¶*On permanent leave from Departamento de Física Aplicada, Universidad de Alicante, 03690 San Vicente del Raspeig, Spain.*

E-mail: joaquin.fernandez-rossier@inl.int

Estimate of resolution

Here we derive the relative error of the temperature measurement method using ESR-STM to determine both the ratio between the peaks and the splitting in the two-level system (TLS) (equation (9) and (10) of the main text). We start with the relation between temperature, ratio, and energy given by $k_BT = -\frac{\varepsilon}{ln(r)}$. We now apply the straightforward formula of error propagation with the two uncertainty sources, r and ε

$$\Delta k_BT = \left|\frac{\partial k_BT}{\partial \varepsilon}\right|\Delta\varepsilon + \left|\frac{\partial k_BT}{\partial r}\right|\Delta r \tag{1}$$

then, we can trivially obtain

$$\Delta k_BT = \left|\frac{1}{ln(r)}\right|\Delta\varepsilon + \left|\frac{\varepsilon}{r ln^2(r)}\right|\Delta r. \tag{2}$$

The uncertainty in the peak height ratio relates to the relative resolution in the current variations that can be registered. We showed in a previous work[1] that:

$$\Delta r = \frac{\Delta I}{I_0}(1 + r) \tag{3}$$

where I_0 is the base current and ΔI is the minimal current variation that can be registered, that is given by shot noise. In a given time interval Δt, an average of N electrons goes through the STM-surface tunnel junction. Assuming a Poissonian distribution, the variance is given by $\sqrt{N}$.

Therefore $\Delta I = \frac{e\sqrt{N}}{\delta t} = \sqrt{\frac{eI}{\Delta t}}$. We substitute in equation (3) and we have $\Delta r = (1 + r)/\sqrt{N}$ and combined with equation (2), we obtain

$$\Delta k_B T = \frac{\Delta \varepsilon}{|ln(r)|} + \frac{\varepsilon(1 + r)}{r\sqrt{N}|ln^2(r)|}. \tag{4}$$

After some algebra, we arrive at the following expression for the relative error:

$$\frac{\Delta T}{T} = \frac{\Delta \varepsilon}{\varepsilon} + \frac{1}{\sqrt{N}} \frac{1 + r}{r|\ln r|}. \tag{5}$$

We can rewrite this equation in terms of inverse temperature and two-level splitting using $r = e^{-\beta\varepsilon}$:

$$\frac{\Delta T}{T} = \frac{\Delta \varepsilon}{\varepsilon} + \frac{1}{\sqrt{N}} \frac{1 + e^{\beta\varepsilon}}{\beta\varepsilon}. \tag{6}$$

Role of back action

We now discuss the role of back-action of the sensor spin on the occupation of the probe spins. Even if flip-flop interactions are blocked, on account of the small ratio between their magnitude and the energy difference of the states GX and XG, the Ising interaction

$jS_z(s)S_z(p)$ is active, where s and p stands for sensor and probe respectively. A lower bound for j is the dipolar interaction:

$$j = \frac{\mu_0 \mu_B^2 S_s S_p}{4\pi d^3} \tag{7}$$

Hence, the effective Hamiltonian for the probe spin would be:

$$H_{\text{probe}} = g_p \mu_B B_{eff} S_z(p) \tag{8}$$

where

$$B_{eff} = B + \frac{jS_z(s)}{g_p \mu_B} \tag{9}$$

in analogy with the effective Hamiltonian for the sensor spin. Now, the main difference between these two situations is the different dynamics of the sensor and probe spins. The sensor spin is being driven by the AC perturbation, and it is therefore fluctuating in a time scale given by Ω^{-1}. In contrast, as long as the flip-flop interactions are blocked, the probe spin is fluctuating in a much slower time scale, given by T_1.

The average magnetization of the driven sensor spin is given by:[2]

$$\langle S_z(s) \rangle = \langle S_z \rangle_0 \left(1 - \frac{T_1 T_2 \Omega^2}{1 + T_2^2 \delta^2 + T_1 T_2 \Omega^2} \right) \tag{10}$$

and T_1, T_2, Ω are the spin relaxation time, spin decoherence time and Rabi driving force of the sensor spin. Thus, the average effective field acting on the probe is given by

$$\langle B_{eff} \rangle = B + \frac{j \langle S_z(s) \rangle}{g_p \mu_B} \tag{11}$$

At resonance, and in the limit $T_1 T_2 \Omega^2 >> 1$, we have $\langle S_z(s) \rangle \to 0$. However, given that the resonance value $\delta = 0$ can only be achieved a fraction of the instances, on account of the fact that δ depends on the state in which the probe spin is, the effective field of the probe spin is *definitely different* from the external field by an amount *smaller* than $\frac{jS_z(s)}{g_p \mu_B}$. We can

therefore provide the following bound to ε

$$\Delta\varepsilon|_{\text{backaction}} \leqslant \frac{jS_z(s)}{g_p\mu_B} \tag{12}$$

taking the most pessimistic assumption that the average spin of sensor spin is maximal. The relative error is given by the ratio of the external field and the stray field:

$$\frac{\Delta\varepsilon|_{\text{backaction}}}{\varepsilon} \leqslant \frac{B_{\text{stray}}}{B} \simeq 10^{-3} \tag{13}$$

Therefore, the back-action error is clearly larger than the shot-noise limit when it comes to determining $\Delta\varepsilon$.

In principle, back-action error does not depend on the measurement time. However, it is apparent that the detection of much smaller stray fields could be achieved if integration time is increased. Another strategy would be to increase the external field, that would require to operate at larger frequencies.

Validity of the Ising model approximation

In this section, we discuss the validity of equation 8, where we assume the system can be described by an Ising model, neglecting flip-flop terms, so that the eigenstates are product states. Flip-flop terms are present both in the exchange and dipolar interactions. For spins on an MgO surface, exchange interaction decays exponentially,[3] so that it can be ignored for adatoms at a 1nm distance. However, dipolar interactions are certainly present.

We can classify the eigenstates of the Ising model in two groups, with $|S_z| = 1$ and $S_z = 0$. The effect of flip-flop interactions is very different in both manifolds: the doublet with $|S_z| = 0$ is almost degenerate, even at finite field. The figure of merit for the effect of flip-flop interactions is the ratio of the Zeeman splitting of the two states with $S_z = 0$. This splitting can originate from two sources: first, a different g factor. Second, a different local

field, as the sensor spin is subject to the tip field.

In order to assess the validity of the Ising approximation, we compute the overlap of the product states and the true eigenstates of a dipolar spin model. For that matter, we take two spins with $g_1 = 2$, $g_2 = 1.9$ separated at a distance $r = 1\,\mathrm{nm}$. This gives a dipolar interaction of approximately $0.2\,\mu$eV. We compute the infidelity, $1 - \langle \sigma_1 \sigma_2 | \Psi_n \rangle$ of the product states $|\sigma_1, \sigma_2\rangle$ with the eigenstates of the Hamiltonian as a function of the applied field B_z. We take the worst-case scenario where the local field is the same for both spins. The results in Figure S1b show that the infidelity for the states with $S_z = 0$ is larger than the states with $|S_z| = 1$, but in both cases is negligible for $B_z > 0.4$ T.

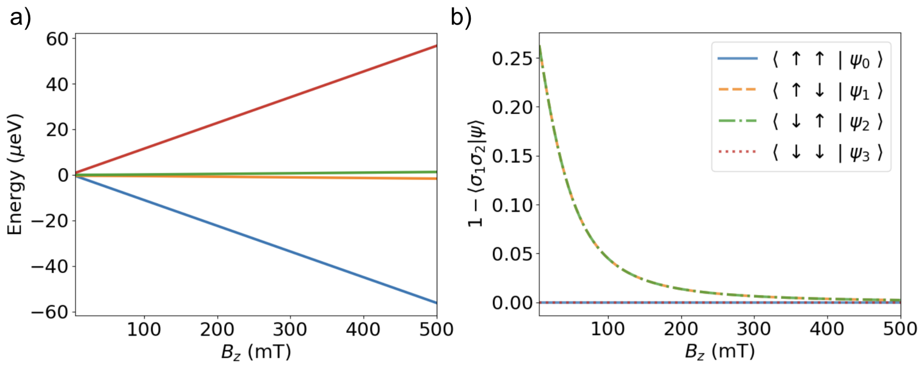

Figure S1: a) Energy level diagram for a dipolar Hamiltonian between two spins separated by 1 nm and with $g_1 = 2$ and $g_2 = 1.9$ as a function of the external magnetic field B_z. b) Fidelity of the states in the system with the product states of two spins as a function of the external magnetic field.

Tip field effect

In this section, we discuss how the tip magnetic field can affect the temperature measurements. The total effective magnetic field on the sensor atom will be the sum of both the external magnetic field and the tip field. The tip field will shift the two resonant peaks in frequency but will not affect their populations. However, the tip field on the probe atom will change its energy splitting.

We can take the experimental measurements for the tip field as a function of the distance

from Yang et al[4] The maximum distance in the experimental data is at 0.45 nm, and the magnetic field is less than 2 mT. Assuming only a few atoms in the tip are responsible for its magnetic moment, if the probe atom is located at 1 nm from the sensor atom, it is safe to assume that the tip magnetic moment will be at least at the same distance. Therefore, it is completely negligible compared to the external magnetic field and other sources of error.

Engineered structures: non-interacting approximation

The probabilities of changing the state of n uncorrelated spins are given by the Boltzmann distribution using the combinatorial formula for $\mathcal{N}$ spins with each possible state where n are excited:

$$P_n(T) = \frac{\frac{\mathcal{N}!}{n!(\mathcal{N}-n)!}e^{-\beta\varepsilon_n}}{\sum_{k=0}^{\mathcal{N}} \frac{\mathcal{N}!}{k!(\mathcal{N}-k)!}e^{-\beta\varepsilon_k}} \tag{14}$$

where ε_n is the energy needed to flip n spins simultaneously. We assume that the energy would be directly proportional to the energy of exciting a single spin, $\varepsilon_n = n\varepsilon_1$.

$$\frac{\Delta T}{T} = \frac{\Delta\varepsilon}{\varepsilon} + \frac{1}{\sqrt{N}}\frac{r+1}{r|\ln{(r/\mathcal{N})}|} = \frac{\Delta\varepsilon}{\varepsilon} + \frac{1}{\sqrt{N}}\mathcal{F}_{\mathcal{N}}(r) \tag{15}$$

Similarly to equation 6, we express the result in terms of $\beta\varepsilon$:

$$\frac{\Delta T}{T} = \frac{\Delta\varepsilon}{\varepsilon} + \frac{1}{\sqrt{N}}\frac{\mathcal{N}+e^{\beta\varepsilon}}{\mathcal{N}\beta\varepsilon} = \frac{\Delta\varepsilon}{\varepsilon} + \frac{1}{\sqrt{N}}\mathcal{F}_{\mathcal{N}}(\beta\varepsilon) \tag{16}$$

Moreover, we notice that for a high number of atoms surrounding the sensor equidistantly, the combinatorial formula may not work since correlations between neighboring spin would decrease the degeneracy for $n > 1$, giving a higher probability to the first excited state, improving the precision at lower temperatures as long as the energy splitting due to neighboring interactions is distinguishable.

Thermal gradient resolution

In this section, we derive the relative temperature difference error (equation (22) in the main text). The temperature difference is given in terms of ε and R_a, R_b:

$$T_{dif} = -\frac{\varepsilon}{k_B}\left(\frac{1}{lnR_a} - \frac{1}{lnR_b}\right) \tag{17}$$

Then, using the error propagation formula, we get:

$$\Delta T_{dif} = \left|\frac{\partial T_{dif}}{\partial \varepsilon}\right| \Delta\varepsilon + \left|\frac{\partial T_{dif}}{\partial R_a}\right| \Delta R_a + \left|\frac{\partial T_{dif}}{\partial R_b}\right| \Delta R_b \tag{18}$$

Trivially, the term associated with the energy of the TLS gives:

$$\left|\frac{\partial T_{dif}}{\partial \varepsilon}\right| \Delta\varepsilon = \frac{\Delta\varepsilon}{k_B}\left|\frac{1}{lnR_a} - \frac{1}{lnR_b}\right| \tag{19}$$

Analogous to the estimate of resolution in temperature, the terms related to the determination of peak height are as follows:

$$\begin{aligned} \left|\frac{\partial T_{dif}}{\partial R_a}\right| \Delta R_a &= \left|\frac{\varepsilon}{R_a ln^2(R_a)}\right| \Delta R_a \\ \left|\frac{\partial T_{dif}}{\partial R_b}\right| \Delta R_b &= \left|\frac{\varepsilon}{R_b ln^2(R_b)}\right| \Delta R_b \end{aligned} \tag{20}$$

using $\Delta r = (1+r)/\sqrt{N}$ we get the following expression:

$$\Delta T_{dif} = \frac{\Delta\varepsilon}{k_B}\left|\frac{1}{lnR_a} - \frac{1}{lnR_b}\right| + \left|\frac{\varepsilon}{k_B R_a ln^2(R_a)}\right| \Delta R_a + \left|\frac{\varepsilon}{k_B R_b ln^2(R_b)}\right| \Delta R_b \tag{21}$$

and after some rearrangement of the equation, we find

$$\Delta T_{dif} = \frac{\Delta\varepsilon}{k_B\varepsilon}\left|\frac{\beta_b - \beta_a}{\beta_a\beta_b}\right| + \frac{1}{\sqrt{N}\varepsilon k_B}\left(\frac{1+R_a}{R_a\beta_a^2} + \frac{1+R_b}{R_b\beta_b^2}\right) \tag{22}$$

Now, we combine ΔT_{dif} with T_{dif} in terms of $\beta_{a,b}$, $T_{dif} = \frac{1}{k_B}\left(\frac{\beta_b - \beta_a}{\beta_a \beta_b}\right)$. The ratio between both expressions gives us the relative error for temperature differences for the gradient determination method:

$$\frac{\Delta T_{dif}}{T_{dif}} = \frac{\Delta \varepsilon}{\varepsilon} + \frac{\beta_a \beta_b}{\sqrt{N}\varepsilon\left(\beta_b - \beta_a\right)}\left(\frac{1+R_a}{R_a \beta_a^2} + \frac{1+R_b}{R_b \beta_b^2}\right) \tag{23}$$

Using $R_a = e^{-\beta_a \varepsilon}$ and $R_b = e^{-\beta_b \varepsilon}$, analogously to the relative error from equations (9) and (10) in the main text, we get a physical form of the expression:

$$\frac{\Delta T_{dif}}{T_{dif}} = \frac{\Delta \varepsilon}{\varepsilon} + \frac{\beta_a \beta_b}{\sqrt{N}\varepsilon(\beta_b - \beta_a)}\left(\frac{e^{\beta_a \varepsilon}+1}{\beta_a^2} + \frac{e^{\beta_b \varepsilon}+1}{\beta_b^2}\right) \tag{24}$$

and the operational form in terms of the ratios, R_a, R_b:

$$\frac{\Delta T_{dif}}{T_{dif}} = \frac{\Delta \varepsilon}{\varepsilon} + \frac{ln(R_a)ln(R_b)}{\sqrt{N}ln\left(R_b/R_a\right)}\left(\frac{1+R_a}{R_a ln^2(R_a)} + \frac{1+R_b}{R_b ln^2(R_b)}\right). \tag{25}$$

where the second part relates to the determination of the ratio and it only depends on the ratios measured and the shot noise, $\frac{1}{\sqrt{N}}\mathcal{F}_{dif}(R_a, R_b)$.

Lateral resolution of the ESR-STM thermal gradient method

The thermal gradient resolution is ultimately limited by the lateral resolution of ESR-STM. This is determined by the smallest distance at which target atoms a and b can be placed. This distance is constrained by the capability of the ESR-STM active sensor to resolve three different peaks, associated to the states GG, XG and GX. This can only happen if both the frequency shifts $\delta f_{a,b} = g_s \mu_B B_{a,b}$, associated to the stray fields created by atoms a and b, B_a, B_b, as well as their difference, $\delta f_a - \delta f_b$ are larger than the peak linewidth δf. Linewidths as small as 3.6MHz in ESR-STM experiments have been reported.[5] For a sensor with $g = 2$,

$S = 1/2$, the shift of the line is 28MHz per mT. In turn, the magnetic field created by a magnetic moment of $1\mu_B$ at 1nm is $\approx$ 1.9 mT. Thus, if we assume that both atoms a and b and the sensor atom have a magnetic moment of $1\mu_B$, and their separation to the sensor atom is $d+x$ and $d-x$, so that they are separated by a distance $2d$, and we take $d = 1$ nm and $x = 0.3$ nm, we find $2|\delta f_a - \delta f_b| \simeq 64$ MHz, sufficient to resolve both peaks given a resonance width of 3.6 MHz. In combination with a relative temperature difference resolution, $\frac{\Delta T_{diff}}{T_{diff}} = 10^{-1}$ in the range of 1K temperatures, gives ESR-STM thermometry a thermal gradient resolution of 5mK/nm.

References

(1) del Castillo, Y.; Fernández-Rossier, J. Certifying entanglement of spins on surfaces using ESR-STM. *Physical Review B* **2023**, *108*, 115413.

(2) Delgado, F.; Fernández-Rossier, J. Spin decoherence of magnetic atoms on surfaces. *Progress in Surface Science* **2017**, *92*, 40–82.

(3) Yang, K.; Bae, Y.; Paul, W.; Natterer, F. D.; Willke, P.; Lado, J. L.; Ferrón, A.; Choi, T.; Fernández-Rossier, J.; Heinrich, A. J.; others Engineering the eigenstates of coupled spin-1/2 atoms on a surface. *Physical Review Letters* **2017**, *119*, 227206.

(4) Yang, K.; Paul, W.; Natterer, F. D.; Lado, J. L.; Bae, Y.; Willke, P.; Choi, T.; Ferrón, A.; Fernández-Rossier, J.; Heinrich, A. J.; others Tuning the exchange bias on a single atom from 1 mT to 10 T. *Physical Review Letters* **2019**, *122*, 227203.

(5) Baumann, S.; Paul, W.; Choi, T.; Lutz, C. P.; Ardavan, A.; Heinrich, A. J. Electron paramagnetic resonance of individual atoms on a surface. *Science* **2015**, *350*, 417–420.
